# MLMI-2024: The dataset of mosquito larvae microscopic images (*Aedes aegypti* and *Culex quinquefasciatus*) collected from Surabaya, Indonesia

**DOI:** 10.1016/j.dib.2025.111641

**Published:** 2025-05-15

**Authors:** Rizka Wakhidatus Sholikah, Afrida Rohmatin Nuriyah, Annisaa Sri Indrawanti

**Affiliations:** Department of Information Technology, Institut Teknologi Sepuluh Nopember, Surabaya, Indonesia

**Keywords:** *Aedes aegypti*, *Culex quinquefasciatus*, Deep learning, Machine learning, Microscopic image, Mosquito larva classification

## Abstract

Indonesia is a tropical country where mosquitoes are common. There are >100 species of mosquitoes found in Indonesia. Some of these species can carry diseases such as dengue fever, which is caused by the *Aedes aegypti* mosquito, and West Nile virus, which is carried by the *Culex quinquefasciatus* mosquito. Mosquito larvae are found in stagnant or slow-moving water, and identifying the species of larvae is important in medical and veterinary entomology to determine the potential risk of disease. The dataset includes two species of mosquito larvae: *Aedes aegypti* and *Culex quinquefasciatus*, which are divided into four parts: head, abdomen-8, siphon, and full body. This dataset can be useful for medical entomologists to classify mosquito larvae quickly and automatically.

Specifications Table

**Table:** 

Subject	Computer vision and pattern recognition, applied machine learning, entomology and insect science
Specific subject area	Mosquito larvae classification
Data format	Raw
Type of data	Images
Data collection	Mosquito larvae are preserved by soaking in formalin for at least 24 h and then going through a multiple-stage dehydration process with ethanol. After that, the larvae are cleared and put in preparation glass for further processing. The larvae are then sliced into four parts: head, abdomen-8, siphon, and full body. To capture microscopic images of the mosquito larvae, a rear phone camera with a resolution of 64MP is used. The resulting image is then cropped to a size of 3456 × 3456 pixels.
Data source location	All the mosquito larvae are obtained and processed at the Entomology Laboratory, Institute of Tropical Disease, Airlangga University, Indonesia. Latitude and Longitude (−7.2694149, 112.7859168)
Data accessibility	Repository name: MLMI-2024 (Mosquito Larvae Microscopic Images) Data identification number: 10.17632/pgby6jmtp4.1 Direct URL to data: https://data.mendeley.com/datasets/pgby6jmtp4/1

## 1 Value of the Data


 

• The dataset contains microscopic images of two species of mosquitoes: *Aedes aegypti* and *Culex quinquefasciatus*. Each species is represented by four images: the full body, head, 8th abdomen, and siphon of the larvae.
• The dataset can be utilized to develop a classification model for mosquito larvae as part of efforts to control mosquito-borne diseases.
• The dataset can be beneficial for entomologists to automate the detection of mosquito species from the larvae and expedite the manual process.
• The dataset can be used to analyze the classification of mosquitoes based on the full body or specific parts of the larvae.
• The dataset can be valuable for understanding the geographical distribution of mosquitoes by analyzing the result of the classification from several locations, so the researcher can map their geographical spread.

## 2 Background

Surabaya is a city in Indonesia with a tropical climate that provides a suitable habitat for various mosquito species, including those that can transmit infectious diseases. Mosquito-borne diseases such as dengue fever, malaria, Zika virus, and chikungunya present significant public health threats worldwide [1,2]. In 2024, the Ministry of Health of Indonesia reported a 20 % increase in dengue fever cases in Surabaya compared to the previous year, which was attributed to unpredictable rainfall. Effective mosquito surveillance and species identification are essential for vector control and disease prevention. Traditional methods of identification depend on expert entomologists examining the morphological features of mosquitoes under a microscope, a process that is both time-consuming and labor-intensive. To improve automated classification and enhance the efficiency of mosquito species identification, we present a dataset of microscopic images of mosquito larvae collected from Surabaya, Indonesia, comprising two species: *Aedes aegypti* and *Culex quinquefasciatus*.

The dataset was collected to support entomological research and the development of artificial intelligence models for automatic classification of mosquito species. By utilizing computer vision and machine learning or deep learning, this dataset aims to enhance the speed and accuracy of identifying mosquito larvae. This advancement will assist public health initiatives in vector surveillance and control.

## 3 Data Description

Indonesia is a tropical country that is suitable for mosquito habitats. The high density of mosquitoes in Indonesia has led to the spread of many diseases carried by these insects. *Aedes* and *Culex* are two genus that can live anywhere and cause deadly diseases [1]. *Aedes aegypti* is the vector of dengue fever, chikungunya, yellow fever, and zika viruses [2,3]; while *Culex*, especially *Culex quinquefasciatus*, is the vector for lymphatic filariasis disease [4]. The recognition of each species of mosquito can be beneficial in mapping the distribution of the potential spread of mosquito-borne disease. Several studies have been conducted to classify mosquitoes automatically. The automation can be done using the model already trained using the available mosquito dataset.

The mosquito dataset was divided into two categories: mosquitoes and mosquito larvae. The mosquito dataset provides images of adult mosquitoes captured directly by a smartphone camera [5]. There are datasets that offer images of mosquitoes from *Aedes aegypti, Anopheles stephensi*, and *Culex quinquefasciatus* [5]. Another study collected images of *Aedes aegypti, Aedes albopictus*, and *Culex quinquefasciatus* using a Digital Single-Lens Reflex (DSLR) 18 MP camera [6]. The geospatial dataset provides records of the spread of adult mosquitoes from 2014 to 2020 [7]. Another published dataset offers images of the larvae stage captured by a microscope. The dataset provides mosquito larvae images from *Aedes* [3,8], *Anopheles*, and *Culex* genus [8].

This dataset provides microscopic images of mosquito larvae collected using a microscope and smartphone camera. Before taking pictures under the microscope, the larvae are prepared to maintain their integrity during data acquisition and enhance visualization. The mosquito larvae will be preserved using a formalin solution. This will be followed by a multilevel dehydration process to remove moisture from the larvae, and finally, the larvae will be cleared using xylene or xylol. The dataset contains two species: *Aedes aegypti* and *Culex quinquefasciatus*. For each species, the dataset includes full body images, images of the head, the 8th abdomen segment, and the siphon. The proposed dataset is organized into two folders. The first folder is named *Aedes aegypti*, and the second one is named *Culex quinquefasciatus*. Each species folder consists of four subfolders named “full body,” “head,” “abdomen,” and “siphon.” The dataset contains original images without augmentation, with each subfolder containing 100 images. The image has size 3456 × 3456 pixel taken by using camera smart phone with 64MP and microscope. Table 1 shows the sample picture of the mosquito larvae dataset.

**Table 1:** The sample of dataset includes Aedes aegypti and Culex quinquefasciatus for each part: full body, head, the 8th abdominal segment, and siphon.

Folder	Subfolder	Sample Image	Description
*Aedes aegypti*	Full body		The image displays the full body of *Aedes aegypti*, from the head to the siphon. It shows a cut in the 8th abdominal segment, as this is necessary for capturing images only of that segment.
	Head		The image shows only the head of the *Aedes aegypti* larvae, allowing for better observation of this part compared to viewing from the full body.
	The 8th abdomen segment		The image shows the eighth abdominal segment of the *Aedes aegypti* larvae, which contains key morphological features for classifying mosquito larvae species.
	Siphon		The image shows only the siphon of *the Aedes aegypti* larvae to enhance observation compared to a full-body image. The siphon is one of the characteristics of mosquito larvae species, which helps differentiate it from other species.
*Culex quinquefasciatus*	Full body		The image shows the full body from head to siphon of *Culex quinquefasciatus*.
	Head		The image displays only the head of the *Culex quinquefasciatus* species for easier observation.
	The 8th abdomen segment		The image shows only the 8th abdominal segment of *Culex quinquefasciatus*, as the 8th abdomen contains key characteristics for classifying the species.
	Siphon		The image shows only the 8th abdominal segment of Culex quinquefasciatus, as the 8th abdomen contains key characteristics for classifying the species.

## 4 Experimental Design, Materials and Methods

### 4.1 Experimental design

**Fig. 1:**
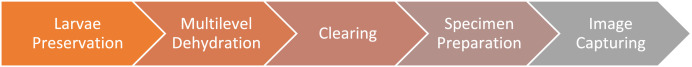
Data acquisition process.

The data acquisition was conducted in collaboration between Institut Teknologi Sepuluh Nopember and the Entomology Laboratory at Airlangga University. The mosquito larvae were collected from the insectarium of the Entomology Laboratory. Mosquito larvae in the insectarium are the result of breeding *Aedes aegypti* and *Culex quinquefasciatus* mosquitoes from endemic areas in the city of Surabaya, Indonesia. The step-by-step process of data acquisition is shown in Fig. 1. The process consists of five main steps: larvae preservation, multilevel dehydration, clearing, specimen preparation, and image capturing. The detailed process will be explained in the following subsection.

### 4.2 Materials

The images were captured using a smartphone with the following specifications:

• Rear camera resolution 64MP
• CPU: Octa-core (2 × 2.0 GHz Cortext-A75 & 6 × 1.8 Ghz Cortex-A-55)
• RAM: 6GB
• Internal Memory: 128 GB

The preparation of mosquito larvae specimens used several materials, including:

• Formalin is used for the preservation of mosquito larvae.
• Ethanol is used in the dehydration process.
• Aquadest is distilled water used to create different concentrations of ethanol.
• Petri dishes are used as containers for the dehydration process.
• Xylene is used in clearing process.
• Microscope slides and cover slips are used to hold larvae for microscope examination.
• Entellan is used to mount the microscope slide and cover slip permanently.

### 4.3 Methods

**Fig. 2:**
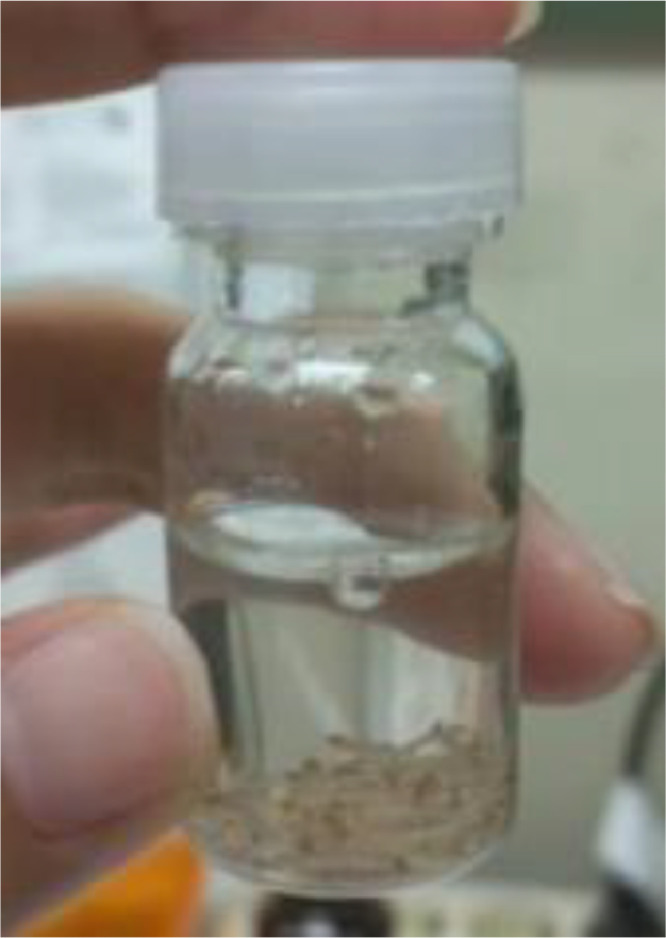
The process of larvae preservation.
(2) Multilevel Dehydration
  Multilevel dehydration is a process used to remove moisture from the larvae. Ethanol is the most commonly used dehydrating agent in the preservation process. The multilevel dehydration process involves using several concentrations of ethanol: 50 %, 70 %, 80 %, 96 %, and 100 %. Each mosquito larvae are soaked, beginning with the lowest concentration and gradually increasing to the highest concentration. For each concentration, it takes around 1 hour. The process of multilevel dehydration is shown in Fig. 3.

(1) Larvae Preparation
  The first step is to preserve the mosquito larvae using a 10 % formalin solution, which requires a minimum of 24 h. The purpose of the preservation is to maintain the integrity of the larvae during the data acquisition process. Fig. 2 depicts the sample of the preservation process.

**Fig. 3:**
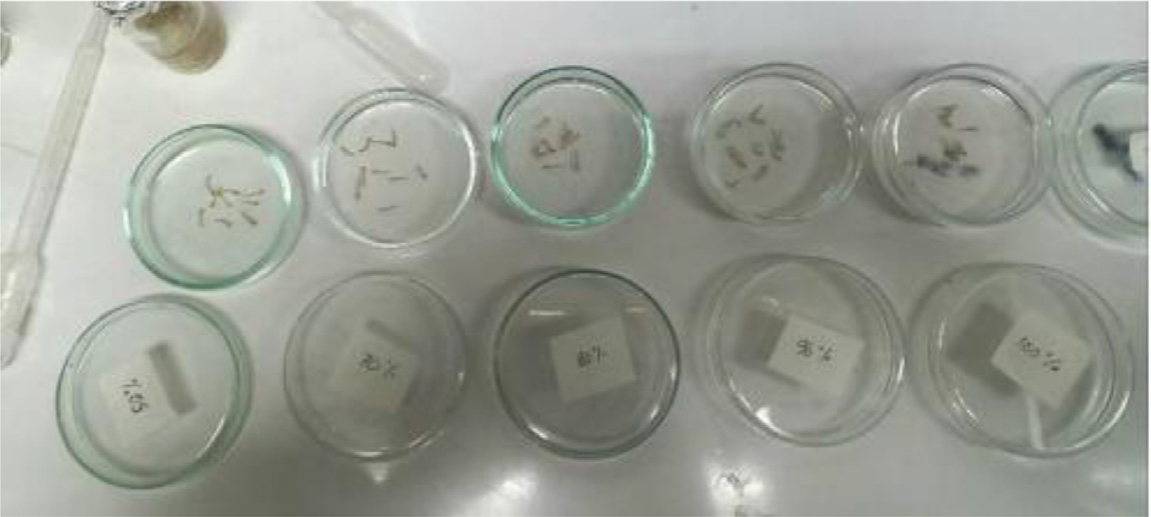
The process of multilevel dehydration.
(3) Clearing
  Clearing is the process of removing substances from inside the larvae, such as ethanol, which is used during the dehydration process. This removal reduces interference during microscopic observation. The clearance process uses xylene or xylol because it can dissolve fats and other substances that are still attached to the mosquito larvae. The clearing process required around 30 min to an hour.
(4) Specimen Preparation
  This procedure is designed to prepare the larvae for observation under the microscope. The first step involves cutting the larvae between the 5th and 6th abdomen, as illustrated in Fig. 4. This is done to facilitate the observation of the 8th segment of the mosquito larva's abdomen. Following the cutting process, the next step is to mount the larvae on a microscope slide and cover slip using Entellan. The mounting process takes one to two days to ensure that it dries perfectly. Once the mounting is complete, the specimens can be used for observation.

**Fig. 4:**
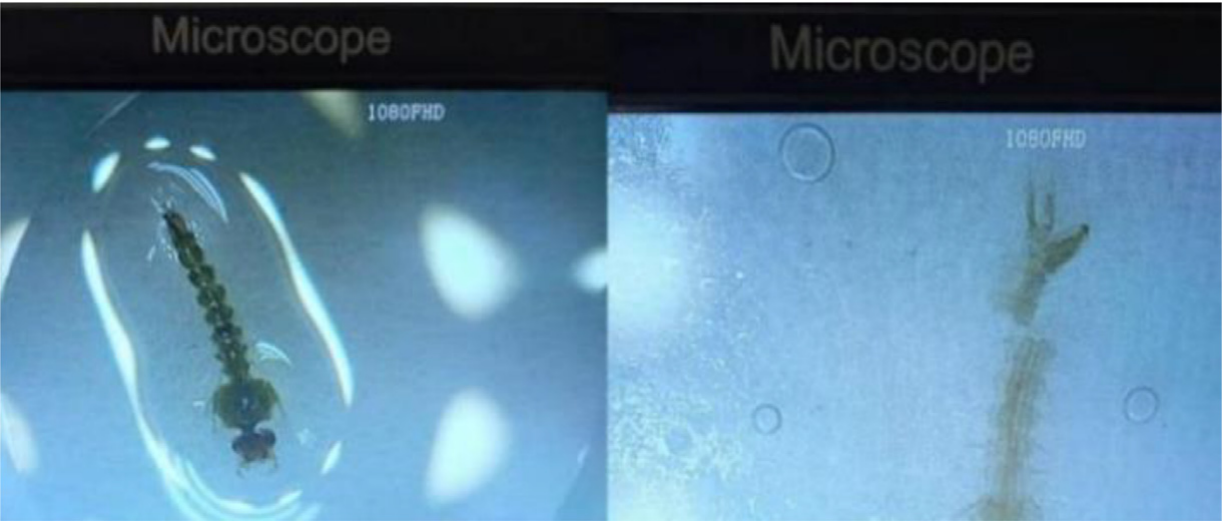
The step of cutting the mosquito larvae abdomen.
(5) Image capturing
  The process of image capturing involves obtaining microscopic images from a microscope. The specimen is observed under the microscope, starting with a full-body observation, then moving on to the head, the 8th segmented abdomen, and the siphon. Microscopic images of each part are captured using the smartphone's rear camera. This process is carried out for both species, *Aedes aegypti* and *Culex quinquefasciatus*. After obtaining the microscopic images, the next step is to store each image in the respective folder. For example, the full-body image of Aedes aegypti is stored in the folder Aedes_aegypti/full_body/imageID.jpg.

## Limitations

None.

## Ethics Statement

This work does not perform any human or animal experiment, or any data collected in social media.

## Credit Author Statement

**Rizka Wakhidatus Sholikah:** Writing – original draft, Conceptualization, Supervision **Afrida Rohmatin Nuriyah**: Resources, Data Curation, Methodology **Annisaa Sri Indrawanti**: Supervision.

## Data Availability

Mendeley DataMLMI-2024 (Original data).
